# H. pylori is related to NAFLD but only in female: A Cross-sectional Study

**DOI:** 10.7150/ijms.50748

**Published:** 2021-04-02

**Authors:** Jingwei Wang, Fengxiao Dong, Hui Su, Licun Zhu, Sujun Shao, Jing Wu, Hong Liu

**Affiliations:** Beijing Shijitan Hospital, Capital Medical University, Beijing, China.

**Keywords:** Female, H. pylori infection, Nonalcoholic fatty liver disease

## Abstract

**Background:** Recently, an increasing number of studies have focused on the extragastrointestinal effects of Helicobacter pylori (H. pylori), including metabolic syndrome, fatty liver, and rheumatic and skin diseases. Nonalcoholic fatty liver disease (NAFLD) is a common chronic disease worldwide that conveys a heavy economic burden on patients and society. The aim of this study was to investigate the relationship between H. pylori and NAFLD and to identify potential influencing factors.

**Methods:** We conducted a cross-sectional study of individuals who had undergone regular physical examinations at the Beijing Shijitan Hospital Health Examination Center from July to October 2018. We evaluated the associations between NAFLD and NAFLD with H. pylori infection and related serum markers using multiple linear regression and logistic regression.

**Results:** There were significant relationships between H. pylori infection status and NAFLD in females (*P*=0.034) but not in males (*P*=0.795) according to Fisher's exact test. The association persisted after further adjustment for metabolic variables, gastrin factors, and liver enzymes. Waist-to-Hip Ratio, Body Mass Index, triglycerides, High-density lipoprotein cholesterol, glucose, uric acid, alkaline phosphatase, and Alanine aminotransferase are related to NAFLD after adjusting for age or interaction between biochemical indexes.

**Conclusion:** H. pylori infection is related to NAFLD in female patients. The relationship between H. pylori and NAFLD may be mediated by markers of lipid metabolism and glycometabolism.

## Introduction

Helicobacter pylori (H. pylori), a Gram-negative, spiral-shaped microaerophilic bacterium, has been proven to be an important pathogen in gastrointestinal diseases 1. Approximately 50% of the world's population has been affected by H. pylori infection, and the number of infected people was 4.4 billion in 2015. H. pylori infection rates vary across different regions, being 50.8% in developing countries and 34.7% in developed countries 2, and approximately 800 million Chinese people are affected by this disease. It may cause chronic inflammation of the gastric mucosa, which may lead to chronic atrophic gastritis, peptic ulcer diseases, and gastric cancer 3, 4. Furthermore, the latest studies have identified associations between H. pylori and extragastrointestinal effects, including metabolic syndrome 5, fatty liver 6, rheumatic and skin diseases 7 and so on. These parenteral diseases associated with H. pylori infection seriously affect patients' general condition and convey a series of complications.

Nonalcoholic fatty liver disease (NAFLD) has been described as a condition of fatty infiltration of the liver in the absence of the other common causes of steatosis, for example, due to high alcohol consumption. The prevalence of NAFLD ranges from 6.3% to 33.0% worldwide 8. Moreover, the prevalence of NAFLD in Asian countries has increased rapidly with a trend toward younger patients during the last two decades. Epidemiological studies show that NAFLD prevalence in the populations of Chengdu (southwest China), Shanghai (east China), Guangdong (south China) and central China is 12.5%, 15.0%, 17.0% and 24.5%, respectively 9. NAFLD includes a series of histological abnormalities, ranging from simple steatosis with traditionally benign clinical process to nonalcoholic steatohepatitis (NASH) characterized by inflammation, fibrosis and occasional cirrhosis. NASH may develop into decompensated liver disease and lead to liver failure, causing heavy economic burdens on both patients and society 9, 10. Therefore, early detection and diagnosis of NAFLD is very important, and it is essential to identify additional risk factors for NFALD morbidity and progression.

Recognized risk factors for NAFLD include insulin resistance, obesity, hypertension, dyslipidemia, type 2 diabetes mellitus and metabolic syndrome 10. Unfortunately, to date, the complex mechanism of NAFLD remains unclear. Recent studies have concentrated their attention on H. pylori and NAFLD 10-12. However, the relationship between these two entities remains controversial. A large-scale cohort study of 17,028 adults was conducted in Korea and verified that H. pylori infection was significantly associated with the development of NAFLD 11. However, another study of 2051 participants concluded that Helicobacter pylori infection did not appear to increase the prevalence of NAFLD 12. Given these discrepancies, more evidence is needed to verify the relationship between these entities. Previous studies have shown that H. pylori infection contributes to development of NAFLD by altering lipid metabolism 11, promoting gastric atrophy 13 and so on. In this study, we performed a cross-sectional investigation to determine the relationship between H. pylori and NAFLD and to identify potential influencing factors.

## Methods

### Study population

**Figure 1:**
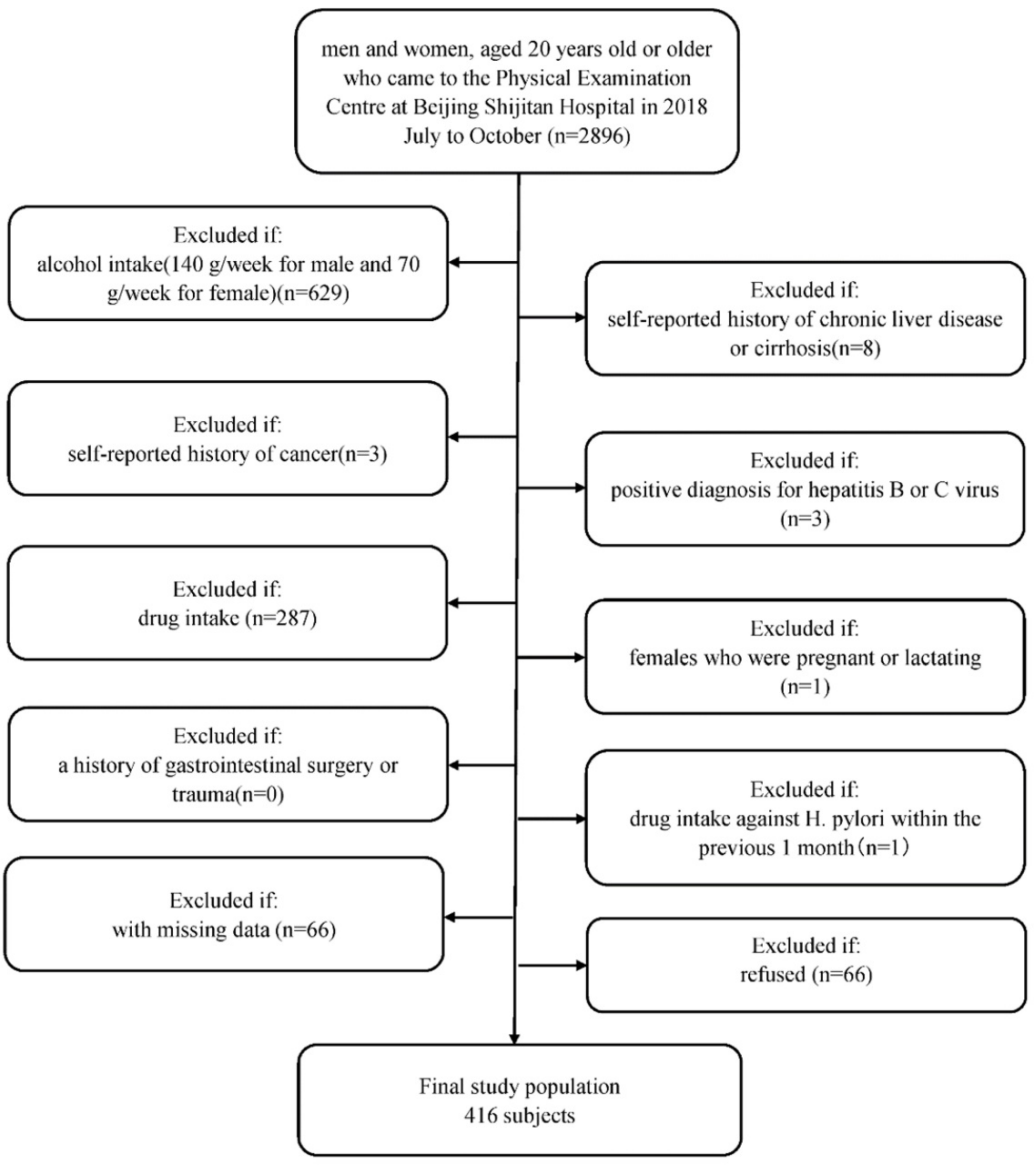
** Flow diagram of study participants.** Flow chart of selection of the included subjects.

Briefly, men and women, aged 20 years old or older, were recruited who regularly had examinations at Beijing Shijitan Hospital Health Examination Center from July to October in 2018. Since we aimed to evaluate the relationship between H. pylori infection and NAFLD, we included a study population who underwent abdominal ultrasonography to assess the degree of steatosis in the liver and H. pylori infection status (n=2896). We excluded patients with (1) alcohol intake (140 g/week for male and 70 g/week for female, n=629); (2) self-reported history of chronic liver disease or cirrhosis (n=8); (3) self-reported history of cancer (n=3); (4) positive diagnosis for hepatitis B or C virus (n=3); (4) drug intake (drugs included antihypertension drugs, antidiabetic drugs, lipid-lowering drugs, corticosteroids, and proton pump inhibitors (n=287); (5) females who were pregnant or lactating (n=1); (6) a history of gastrointestinal surgery or trauma (n=0); and (7) drug intake against H. pylori within the previous 1 month (n=1). Furthermore, we excluded 66 participants with missing data. Finally, 1898 patients agreed to be in the study, and 66 people refused. A MOOSE flow chart is shown in Figure 1. This study was approved by the Ethics Committee of Beijing Shijitan Hospital Affiliated to Capital Medical University and was performed in accordance with the Declaration of Helsinki. Written informed consent was obtained from all participants.

### Data collection

Questionnaires were completed by all study participants, including demographics (age, sex, race), smoking status, alcohol consumption, medicine use and personal medical history (obesity, hypertension, dyslipidemia, type 2 diabetes mellitus, metabolic syndrome, cancer). All blood measurements were performed with fresh serum obtained after a 12-hour fast to minimize the confounding effects of diurnal variation on hormone concentrations and included glucometabolic markers, liver function, renal function, lipid metabolism, ions (calcium, iron), tumor markers, pepsinogen (PG), and pro-gastrin-releasing peptide (proGRP). Anthropometric measurements, including waist circumference (cm), blood pressure (mmHg), body weight (kg) and height (cm), were measured by trained nurses using a standardized protocol. In addition, diastolic and systolic blood pressure were measured in the morning. Body mass index (BMI) was calculated by taking a person's weight, kilograms, divided by their height, in meters squared. Waist-to-hip ratio (WHR) was measured as waist circumference divided by hip circumference.

H. pylori infection status was measure by ^13^C breathing test on the same day in the morning when subjects had an empty stomach. The procedure was performed as follows: (1) two collecting bags were labeled with patient ID; (2) the air exhaled by the patient when they are breathing quietly was collected in one bag as the 0-minute sample; (3) the patient was given a urea (^13^C) capsule with 50 ml water, and 30 min later, the quiet breathing air was collected in the second bag (HCBT-01 tester; Shenzhen Zhonghe Headway Biological Technology Co. Ltd., China). The result is shown as delta over baseline (DOB), which indicates the ratio of ^13^CO2/^12^CO2 with the metabolic activity induced by labeled urea. A DOB greater than 4% is considered an active infection.

Diffuse fatty liver can be defined by the presence of at least two of three abnormal findings on abdominal ultrasonography: diffusely increased liver near field ultrasound echo ('bright liver'), liver echo greater than kidney; vascular blurring and the gradual attenuation of far field ultrasound echo 14. NAFLD patients were confirmed by the following criteria: (1) diffuse fatty liver in ultrasound; (2) no significant alcohol consumption (<140 g (male) or <70 g (female) alcohol consumption per week); and (3) no coexisting causes of chronic liver disease, such as hepatitis C, medication, parenteral nutrition, Wilson's disease or severe malnutrition 15. Participants with fatty liver were divided into mild, moderate and severe categories according to the ultrasonic findings. Steatosis is graded as follows: Absent (score 0) when the echotexture of the liver is normal; mild (score 1), when there is a slight and diffuse increase of liver echogenicity with normal visualization of the diaphragm and of the portal vein wall; moderate (score 2), in case of a moderate increase of liver echogenicity with slightly impaired appearance of the portal vein wall and the diaphragm; severe (score 3), in case of marked increase of liver echogenicity with poor or no visualization of portal vein wall, diaphragm, and posterior part of the right liver lobe 16. All ultrasonography was performed by two professional ultrasound doctors who were uniformly trained to diagnose fatty liver.

The study population was further divided into the risk and nonrisk groups. The risk group was defined as the simultaneous occurrence of one or more of the following risk factors: (1) FPG ≥ 6.10 mmol/L; (2) TG ≥ 1.70 mmol/L; (3) TC ≥ 5.20 mmol/L; (4) HDL-C < 1.00 mmol/L; (5) LDL-C ≥ 3.10 mmol/L; (6) BMI ≥ 24.90 kg/m^2^; (7) waist circumference ≥ 90 cm, triglyceride levels ≥ 150 mg/dL, HDL cholesterol ≤ 40 mg/dL, systolic blood pressure ≥ 130 mmHg, diastolic blood pressure ≥ 85 mmHg, FBG levels ≥ 100 mg/dL (satisfied more than three). Those who did not satisfy the risk criteria were allocated into the nonrisk group 11.

### Statistical analyses

We used SPSS statistical software version 22.0 for data analyses. Continuous variables are reported as the means ± standard deviation, whereas categorical variables are presented as percentages. The Kolmogorov-Smirnov test was used to verify whether the data was normally distributed, and all continuous variables that did not conform to a normal distribution underwent transformation for analysis. Study subjects were classified into H. pylori positive and H. pylori negative according to H. pylori infection status, and summary and grouping data for baseline characteristics (the laboratory examination) were compared using t-test for continuous variables and Fisher's exact test for categorical variable, which is shown in Table 1.

Multivariate-adjusted odds ratios (ORs) and 95% confidence intervals (CIs) were calculated using logistic regression in the mild, moderate and severe fatty liver groups. A logistic regression model adjusted for age or interaction between biochemical indexes was used to assess associations between NAFLD and H. pylori infection status by dichotomizing factor levels and calculating the odds ratios (ORs).

We fit additional models adjusted for metabolic factors, gastrin factors and liver function factors that could be potential confounders or mediators of the association between H. pylori infection and incident NAFLD. Model 1 was adjusted for confounding factors and fasting blood glucose, Ghb, TC, TG, LDL-C, HDL-C, and UA; model 2 was further adjusted for BMI, WHR, SBP, and DBP; model 3 was further adjusted for AST, ALT, and GGT; Finally, model 4 was adjusted for model 3 plus PGI, PGII, PGI/PGII, and proPG.

To further analyze the relationship between H. pylori infection and NAFLD, we analyzed the relationship between the risk and nonrisk group populations based on the degree of NAFLD. All models excluded age as a confounder. P-value < 0.05 was considered statistically significant.

## Results

Data were analyzed from 1898 patients, age 22 to 67 years old, who were examined at Beijing Shijitan Hospital Health Examination Center. The baseline characteristics of the participants are shown in Table 1. The mean participant age was 37.19±0.170, 1217 (64.1%) were male, and 681 (35.9%) were female. Among the study population, 25 (1.3%) participants had severe NAFLD, 174 (9.2%) had moderate NAFLD, 306 (16.1%) had mild NAFLD and 1393 (73.4%) did not have NAFLD. The mean age of the NAFLD and non-NAFLD groups was 36.42±7.031 and 39.31±8.010, and this difference was statistically significant (t=-7.154, *P*<0.001). There was also a significant difference between male and the female participants (*P*<0.001), which is shown in Table 2.

Among all patients, 689 participants presented with H. pylori infection, and 1209 participants did not have an H. pylori infection. The mean age of patients with and without H. pylori infection was 37.04±0.289 (66.5% male), 37.28±0.210 (62.8% male). Table 1 shows that UA (t=-2.082, *P*=0.038) and HDL-C (t=-2.449, *P*=0.014) were significantly different between those with H. pylori infection and those without H. pylori infection, but there was no significant difference between H. pylori infection status and NAFLD.

In Table 2, by separating the study population according to sex, we found that there was a significant relationship between H. pylori infection status and NAFLD in female patients (*P*=0.034) but not in male patients (*P*=0.795).

As shown in Table 3, in a separate logistic regression, there was a significant positive association of WHR (OR=0.041, *P*<0.001), BMI (OR=0.032, *P* <0.001), TG (OR=0.087, *P* <0.001), HDL-C (OR=0.086, *P* <0.001), GLU (OR=0.086, *P* <0.001), UA (OR=0.138, <0.001), ALP (OR=0.124, *P* =0.004), ALT (OR=0.124, *P* =0.004) and C13 (OR=0.473, *P* =0.007) with NAFLD after adjusting for age in female patients. Furthermore, WHR (OR=0.097, *P* <0.001), BMI (OR=0.074, *P* <0.001), TG (OR=0.165, *P* <0.001), HDL-C (OR=0.309, *P* <0.001), GLU (OR=0.197, *P* =0.042), UA (OR=0.240, *P* <0.001) and ALP (OR=0.144, *P* =0.008), and ALT (OR=0.076, *P* =0.001) were significantly associated in the biochemical indexes model in female patients with NAFLD.

To explore whether the increased incidence of NAFLD with H. pylori infection was mediated by metabolic risk factors, gastrin factors or liver enzymes, we conducted additional analyses adjusted for potential mediators in female participants (Table 4). After adjusting for metabolic variables, such as fasting blood glucose, Ghb, TC, TG, LDL-C, HDL-C, and UA (Model 3), the association between H. pylori infection and incident NAFLD was attenuated but remained statistically significant, suggesting mediation by these metabolic factors.

We next separated the study population into risk and nonrisk groups among female participants, which is presented in Table 5. In the risk group, 25 subjects were classified as having moderate NAFLD, 26 subjects as mild NAFLD and 146 as normal liver. In the nonrisk group, 1 subject had severe NAFLD, 3 subjects had moderate NAFLD, 6 subjects had mild NAFLD and 474 had normal liver. We analyzed the relationship between H. pylori infection and the risk/nonrisk groups, observing a significant difference between H. pylori infection (OR=0.502, 95% CI 0.263-0.961 *P*=0.037) and the female participants with risk. There was a trend that did not reach significance between H. pylori infection (OR=0.512, 95% CI 0.083, 1.104 *P*=0.037) and the nonrisk group in female participants. All analyses excluded age as a confounder.

## Discussion

NAFLD is an important health and societal burden in Asian people, both females and males. There are numerous NAFLD-related risk factors, including insulin resistance, obesity, hypertension, dyslipidemia, type 2 diabetes mellitus and metabolic syndrome. Therefore, we conducted a cross-sectional study to identify risk factors for NAFLD.

In this study, we found that H. pylori infection is a risk factor for NAFLD in female patients. After further adjustment of the model for metabolic risk factors, liver enzymes and pepsin related factors, this association persisted. Adjustment for metabolic variables, such as fasting blood glucose, Ghb, TC, TG, LDL-C, HDL-C, and UA, reduced the association between H. pylori infection and NAFLD, but the association was still statistically significant, verifying our result. However, the relationship between H. pylori and NAFLD remains controversial. A large-scale cohort study in Korea contends that H. pylori infection is significantly associated with the development of NAFLD, independent of metabolic and inflammatory risk factors 11. Another meta-analysis including 13 observations concluded that H. pylori infection is associated with NAFLD in middle-aged individuals 17. However, another large-scale cross-sectional study in Japan reported that H. pylori infection was not associated with fatty liver disease 18. In China, a cross-sectional study including 21456 participants reported that H. pylori infection was not independently associated with the risk of NAFLD in apparently healthy subjects 19. The heterogeneity of research results among different studies may be caused by different sample sizes, sample baseline characteristics, Mets diagnostic criteria, follow-up time differences and exclusion of different confounding factors.

Our research found that H. pylori are related to NAFLD only in female patients, which we believe is for the following reasons. First, due to the protection afforded by sex hormones in women, premenopausal women are less likely to suffer from NAFLD than men 20. NAFLD guidelines also state that being male is a risk factor for NAFLD; therefore, we believe that there are differences between men and women with respect to the factors that influence NAFLD. Second, due to cultural factors, people tend to conceal their alcohol intake history. Although we repeatedly stressed the importance of the participants revealing the amount of alcohol they drank, we cannot exclude the possibility of concealment of daily alcohol intake by some participants, which may lead to biased results. Meanwhile, female participants usually drink less than men, so this effect will likely be smaller in women. Last but not least, essays support the idea that men are more likely to suffer from secondary diseases, for example, rheumatoid arthritis, alcoholism, excessive smoking, and gonadal deficiencies, among others 21, which may predispose to NAFLD. The mechanism of NAFLD is very complex and is not entirely clear at present. Our study demonstrated that NAFLD is related to H. pylori in female, but not male, patients. We suspect this might be due to differences in the etiology of NAFLD and influencing factor between male and female patients. However, we did not find any literature to support this connection, which needs additional systematic research to confirm.

In male participants, we found that BMI, WHR, ALT, UA, TG, HDL-C, and Ghb were related to NAFLD. In female participants, we found that BMI, WHR, ALT, UA, GLU, ALP, and C13 were related to NAFLD. TG and HDL-C had a trend toward a significant difference in female participants, indicating that these metabolic factors may be intermediates in the relationship between H. pylori and NAFLD. There is increasing evidence that NAFLD is associated with obesity, dyslipidemia, diabetes and insulin resistance and is considered to be a liver manifestation of metabolic syndrome 18, which is supported by our results.

After excluding age, sex, systolic blood pressure, TC, TG, LDL-C, HDL-C, ALT, AST, GGT as confounders, a significant association between H. pylori and NAFLD persisted. Although the relationship between H. pylori and NAFLD still needs further study, experts think the following reason may explain the connection. First, H. pylori infection is thought to increase the rate of metabolic syndrome 22, 23. Seon Hee Lim conducted a multicenter study in Korea and found that H. pylori infection plays an independent role in the pathogenesis of metabolic syndrome. Furthermore, metabolic syndrome is thought to be related to NAFLD 24, which is supported by our results as well. In addition, H. pylori infection may influence lipid metabolism, including low HDL-C, high LDL-C and TG 25-27, as well as abnormal glycometabolism, including increased fasting glucose and insulin levels 28. Our research revealed significant differences in TG and HDL-C in different degrees of fatty liver patients, and we got same result as the previous research 11. Second, Helicobacter pylori infection may induce NAFLD through transmission of inflammatory stimuli (e.g., lipopolysaccharide (LPS)) directly into the portal venous circulation. H. pylori infection has also been shown to exert systemic effects through the release of vasoactive substances and inflammatory cytokines (e.g., by increasing levels of interleukin-6, tumor necrosis factor-alpha and other proinflammatory cytokines), which may play an important role in the development of NAFLD by enhancing oxidative stress, promoting the transition to adipogenesis and increasing the chance of insulin resistance 6, 29-34. Last but not least, gastric atrophy caused by Helicobacter pylori may lead to gastric acid loss, subsequent small intestinal bacterial overgrowth (SIBO), and increased intestinal permeability, which may increase portal endotoxins 13, 33, 35. Cytotoxin associated gene A antigen (CagA), the virulence factor of H. pylori, has been proven to alter the gut microbiota, resulting in exacerbation of cell proliferation and immune phenotypes 36. Although H. pylori infection may have a beneficial effect on serum liver enzyme levels and other surrogate markers of NAFLD, the effect of early eradication therapy is still insufficient. The limited available research indicates that H. pylori eradication therapy improves nonalcoholic fatty liver disease fibrosis score and may led to greater improvement in HOMA-IR 37, 38.

Despite these relevant findings, our study has several limitations. First, because most patients cannot remember when they were first diagnosed with H. pylori infection, we were not able to obtain the time of H. pylori infection, so different infection times may have had an impact on the results. Second, the sample size of our data was not large enough, and the study population only included participants from Beijing Shijitan Hospital, meaning that there might have been confounding factors due to differences in the distribution of hospital study populations. Third, we did not obtain a study population with successful eradication of H. pylori infection. Additional large-scale studies in the general population are needed to validate our results. Fourth, we only used ultrasound as a criterion for the diagnosis of fatty liver; however, the ultrasound results were completed by two professional ultrasound doctors, which we think were accurate. Fifth, we did not measure participant study indicators repeatedly. In addition, we identified some differences between men and women but failed to further explore them.

In conclusion, this study demonstrates that H. pylori infection is related to NAFLD in female patients. This effect might be exerted through markers of lipid metabolism and glycometabolism. Considering the limited clinical data in this study, it is necessary to further investigate the relationship between H. pylori infection and NAFLD, which undoubtedly will provide new insight for effective prevention and treatment of NAFLD.

## Figures and Tables

**Table 1:** Baseline characteristics of the patients according to the H. pylori infection statue

	Total	H. pylori +	H. pylori -	*P*-value
Age	37.19±0.170	37.04±0.289	37.28±0.210	0.452
**Sex**				
Female	35.9%	33.5%	37.2%	0.107
Male	64.1%	66.5%	62.8%	
SP	114.26±0.330	114.68±0.574	114.02±0.401	0.476
BP	68.400±0.244	68.580±0.417	68.300±0.301	0.894
BMI	23.165±0.078	23.229±0.126	23.114±0.099	0.300
WHR	0.873±0.001	0.873±0.002	0.873±0.002	0.533
ALT	22.861±0.207	23.087±0.606	22.732±0.470	0.260
AST	19.856±0.207	20.255±0.355	19.628±0.255	0.156
UA	360.730±2.099	366.520±3.427	357.440±2.650	**0.019**
TC	4.562±0.019	4.559±0.032	4.563±0.023	0.836
TG	1.345±0.022	1.410±0.044	1.308±0.025	0.210
HDL-C	1.306±0.007	1.285±0.010	1.318±0.008	**0.024**
LDL-C	2.408±0.013	2.409±0.227	2.408±0.017	0.985
GLU	4.714±0.020	4.760±0.038	4.687±0.223	0.348
ALP	59.660±0.454	60.020±0.630	59.450±0.615	0.188
GGT	24.620±0.607	26.060±1.350	23.800±0.561	0.397
PGI (ng/ml)	48.630±24.077	55.317±20.816	44.819±24.971	**0.000**
PG2 (ng/ml)	8.361±6.275	11.051±6.151	6.827±5.815	**0.000**
PG1/PG2	6.437±1.800	5.633±1.810	6.895±1.626	**0.000**
proGRP (pg/mL)	28.080±0.210	28.194±0.375	28.015±0.252	0.952
Ghb (%)	5.379±0.013	5.406±0.025	5.364±0.134	0.676

Abbreviations as in Table 1 and Table 2; OR, odds ratio; Bold indicates statistically significant values. BMI, Body Mass Index; WHR, waist-to-hip ratio; TC, total cholesterol; Ghb, glycosylated hemoglobin; TG, triglyceride; UA, uric acid; AST, Aspartate aminotransferase; ALT, Alanine aminotransferase; Ca, calcium; DP, diastolic blood pressure; SP, Systolic pressure; Hb, hemoglobin; HDL-C, High-density lipoprotein cholesterol; LDL-C, Low-density lipoprotein cholesterol; UA, Uric acid; GLU, glucose; PG, pepsinogen; proGRP, Pro-gastrin-releasing Peptide; ALP, alkaline phosphatase;GGT, γ-glutamyl transpeptidase.

**Table 2:** The relationship between the H. pylori infection and the NAFLD level in different genders

	H. pylori infection (-)	H. pylori infection (+)	*P*-value
**Female**			
Normal	418 (92.9%)	202 (87.4%)	**0.034**
mild NAFLD	19 (4.2%)	13 (5.6%)	
moderate NAFLD	13 (2.9%)	15 (6.5%)	
severe NAFLD	0 (0.0%)	1 (0.4%)	
**Male**			
Normal	485 (63.9%)	288 (62.9%)	0.795
mild NAFLD	168 (22.1%)	106 (23.1%)	
moderate NAFLD	93 (12.3%)	53 (11.6%)	
severe NAFLD	13 (1.7%)	11 (2.4%)	

Bold indicates statistically significant values.

**Table 3:** The relationship between NAFLD statue and the potential biomarker

Characteristics	Model 1†	Model 2‡	Characteristics	Model 1†	Model 2‡
	OR (95% CI)	OR (95% CI)		OR (95% CI)	OR (95% CI)
**WHR**			**BMI (kg/m^2^)**		
Q1	0.041 (0.012,0.133)	0.378 (0.285,0.503)	Q1	0.032 (0.016,0.064)	0.201 (0.151,0.268)
Q2	0	0	Q2	0	0
*P*-value	**0.000**	**0.000**	*P*-value	**0.000**	**0.000**
**SP (mmHg)**			**BP (mmHg)**		
Q1	0.284 (0.047,1.726)	0.719 (0.352,1.467)	Q1	0.061 (0.007,0.553)	0.656 (0.313,1.376)
Q2	0	0	Q2	0	0
*P*-value	0.172	0.364	*P*-value	**0.013**	0.265
**TC (mmol/L)**			**TG (mmol/L)**		
Q1	0.359 (0.176,0.733)	1.758 (0.607,5.099)	Q1	0.087 (0.045,0.169)	0.165 (0.076,0.355)
Q2	0	0	Q2	0	0
*P*-value	**0.005**	0.299	*P*-value	**0.000**	**0.000**
**HDL-C (mmol/L)**			**LDL-C (mmol/L)**		
Q1	5.830 (2.959,11.484)	0.309 (0.110,0.868)	Q1	0.605 (0.154,2.377)	0.384 (0.122,1.208)
Q2	0	0	Q2	0	0
*P*-value	**0.000**	**0.026**	*P*-value	0.472	0.102
**GLU (mmol/L)**			**Ghb (%)**		
Q1	0.086 (0.023,0.321)	0.197 (0.041,0.942)	Q1	0.711 (0.177,2.849)	0.342 (0.096,1.219)
Q2	0	0	Q2	0	0
*P*-value	**0.000**	**0.042**	*P*-value	0.630	0.098
**UA (umol/L)**			**ALP (U/L)**		
Q1	0.138 (0.075,0.252)	0.240 (0.120,0.479)	Q1	0.124 (0.030,0.514)	0.144 (0.035,0.599)
Q2	0	0	Q2	0	0
*P*-value	**0.000**	**0.000**	*P*-value	**0.004**	**0.008**
**GGT (U/L)**			**ALT (U/L)**		
Q1	0.409 (0.078,2.158)	2.497 (0.294,21.221)	Q1	0.057 (0.017,0.195)	0.076 (0.016,0.357)
Q2	0	0	Q2	0	0
*P*-value	0.292	0.402	*P*-value	**0.000**	**0.001**
**AST (U/L)**			**PGI (ng/ml)**		
Q1	0.069 (0.014,0.345)	0.420 (0.045,3.896)	Q1	1.857 (1.064,3.245)	1.597 (0.786,3.245)
Q2	0	0	Q2	0	0
*P*-value	**0.001**	0.445	*P*-value	**0.029**	0.196
**PGII (ng/ml)**			**PGI/PGII**		
Q1	1.252 (0.721,2.175)	0.962 (0.432,2.140)	Q1	1.266 (0.736,2.179)	1.212 (0.628,2.335)
Q2	0	0	Q2	0	0
*P*-value	0.424	0.923	*P*-value	0.393	0.567
**proPG (pg/ml)**			**C13**		
Q1	1.266 (0.736,2.179)	1.212 (0.628,2.335)	Q1	0.473 (0.274,0.817)	-
Q2	0	0	Q2	0	
*P*-value	0.393	0.567	*P*-value	**0.007**	

Abbreviations as in Table 1; OR, odds ratio; The model 1† only used age as a covariable to the logistic regression models; In the model 2 ‡, the variables were divided into four groups: basic information (WHR, BMI, SP, BP); metabolic index (TC, TG, HDL-C, LDL-C, GLU, Ghb, UA), Liver related indicators (ALP, GGT, ALT) and gastrin related indexes (PGI, PGII, PGI/PGII, proPG). When analyzed one of the variables, other variables in the same group and age were included in the model as covariables; Bold indicates statistically significant.

**Table 4:** Mediation analysis of the association between H.pylori status and the development of nonalcoholic fatty liver disease (NAFLD)

	OR	95%CI	*P*
Model 0, aHRa (95% CI)	0.473	0.274, 0.817	0.007
Model 1, aHRa (95% CI)	0.517	0.274, 0.974	0.041
Model 2, aHRa (95% CI)	0.395	0.164, 0.949	0.038
Model 3, aHRa (95% CI)	0.395	0104, 0.807	0.018
Model 4, aHRa (95% CI)	0.221	0.067, 0.736	0.014

Model 0: adjusted for age; Model 1: Model 0 plus adjustment for fasting blood glucose, Ghb, TC, TG, LDL-C, HDL-C, UA; Model 2: Model 1 plus adjustment for BMI, WHR, SBP, and DBP; Model 3: Model 2 plus adjustment for AST, ALT, and GGT; Model 4: Model 3 plus adjustment for PGI, PGII, PGI/PGII, proPG.

**Table 5:**
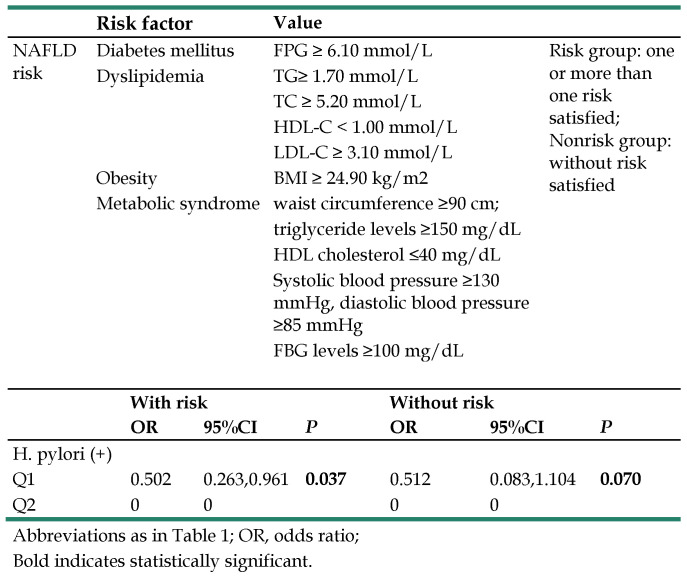
Definition of NAFLD risk group and the relationship between H. pylori infection and NAFLD in risk and non-risk group

## Abbreviations

PG): Lumbar dual-energy X-ray absorptiometry (DXA
ALP): alkaline phosphatase
GGT: γ-glutamyl transpeptidase
